# Differences in gene expression patterns between cultured and natural *Haloquadratum walsbyi* ecotypes

**DOI:** 10.3389/fmicb.2022.1044446

**Published:** 2022-11-10

**Authors:** Riccardo Rosselli, Mario López-Pérez, Ana-Belen Martin-Cuadrado, Francisco Rodriguez-Valera, Henk Bolhuis

**Affiliations:** ^1^Department of Marine Microbiology and Biogeochemistry, Royal Netherlands Institute for Sea Research (NIOZ), Den Hoorn, Netherlands; ^2^Department of Physiology, Genetics and Microbiology, University of Alicante, Alicante, Spain; ^3^LABAQUA S.A., Research & Development Department, Las Atalayas, Alicante, Spain; ^4^Evolutionary Genomics Group, División de Microbiología, Universidad Miguel Hernández, San Juan de Alicante, Spain

**Keywords:** RNA-seq, metatranscriptome, solar saltern, archaea, *Haloquadratum walsbyi*

## Abstract

Solar crystallizer ponds are characterized by high population density with a relatively simple community structure in terms of species composition. The microbial community in the solar saltern of Santa Pola (Alicante, Spain), is largely dominated by the hyperhalophilic square archaeon *Haloquadratum walsbyi*. Here we studied metatranscriptomes retrieved from a crystallizer pond during the winter of 2012 and summer of 2014 and compared *Hqr. walsbyi’s* transcription patterns with that of the cultured strain *Hqr. walsbyi* HBSQ001. Significant differences were found between natural and the cultured grown strain in the distribution of transcript levels per gene. This likely reflects the adaptation of the cultured strain to the relative homogeneous growth conditions while the natural species, which is represented by multiple ecotypes, is adapted to heterogeneous environmental conditions and challenges of nutrient competition, viral attack, and other stressors. An important consequence of this study is that expression patterns obtained under artificial cultivation conditions cannot be directly extrapolated to gene expression under natural conditions. Moreover, we found 195 significantly differential expressed genes between the seasons, with 140 genes being higher expressed in winter and mainly encode proteins involved in energy and carbon source acquiring processes, and in stress responses.

## Introduction

Microbial communities in hypersaline crystallizer ponds have been extensively studied for over 50 years (Oren, 1994). Whereas initial research focused on the physiological characterization of halophilic bacterial and archaeal isolates, developments in high-throughput nucleotide sequencing strongly reduced cultivation efforts. Initial analyzes of the 16S rRNA gene through Sanger sequencing (Benlloch et al., 1995) provided insight in the taxonomic diversity, while current metagenomic approaches address the genetic potential of halophiles in crystallizers around the world (Legault et al., 2006; Sanchez-Perez et al., 2008; Narasingarao et al., 2011; Plominsky et al., 2014; Naghoni et al., 2017). However, these techniques provide little information about physiological activities of halophiles in their natural environments. A high-resolution approach to deduce active microbial processes in natural habitats is metatranscriptomics as the analysis of transcribed messenger RNA (mRNA) provides information on which genes are actively expressed under extant conditions. Although a one-to-one relationship between gene expression and protein synthesis (or enzymatic activity) is not always evident due to post-transcriptional (Fu et al., 2013; Peterson et al., 2016) and translational modifications (Redon et al., 2005; Li et al., 2014), metatranscriptomics offer a good insight into gene regulation and environmental responses.

In contrast to experiments with monocultures growing under well-defined laboratory conditions, natural communities are exposed not only to numerous environmental fluctuations (light, dark, cold, hot, rain, desiccation, etc.) but also to interactions with other microorganisms and with bacteriophages. Examples of interspecies effects on gene expression patterns are the mutualistic interactions between the marine bacteria *Prochlorococcus* and *Alteromonas macleodii* (Kimes et al., 2014; Aharonovich and Sher, 2016; Biller et al., 2016) between *Synechococcus* sp. PCC7002 and *Shewanella putrefaciens* (Beliaev et al., 2014). Transcriptomics can also reveal antagonistic interactions such as found between the two fish-pathogens, *Moritella viscosa* and *Aliivibrio wodanis* (Hjerde et al., 2015). Interestingly, also within a single species, competition between closely related strains of *Salinibacter ruber* (M8 and M31) revealed a significant change in their transcription patterns when growing in mono- or coculture (Gonzalez-Torres et al., 2015). Transcriptomics was also used to uncover expression of potential mating factors in two near identical mutants of *Haloferax volcanii* (Makkay et al., 2020). Especially genes encoding glycosylation proteins and those with unknown function were overexpressed under mating conditions. Along these lines, several antagonistic interactions were described among extremely halophilic prokaryotes due to the production of antimicrobial metabolites such as halocins (Atanasova et al., 2013; Ghanmi et al., 2016). We therefor need to be aware that gene expression patterns of key species under laboratory conditions may not necessarily be directly translated to the natural behavior of these species.

Here we analyzed and compared gene expression patterns in the cultivated pure culture of *Hqr. walsbyi* strain HBSQ001 with that of natural occurring *Hqr. walsbyi ecotypes* in the crystallizer pond CR30 of the Bras del Port saltern, Santa Pola (Alicante, Spain). *Hqr. walsbyi* is notorious for its peculiar square morphology and largely dominates the crystallizer (Bolhuis et al., 2004) with numbers up to 10^8^ cells per ml and constituting up to 80% of the total microbial population (Benlloch et al., 2001; Ghai et al., 2011). This crystallizer basin is the same from which strain HBSQ001 was originally isolated (Bolhuis et al., 2004) and has been the focus of a series of molecular studies over the last 25 years, and substantial information regarding its microbial composition is well documented (Benlloch et al., 1995; Martínez-Murcia et al., 1995; Benlloch et al., 1996; Antón et al., 1999; Benlloch et al., 2002; Øvreås et al., 2003; Papke et al., 2004; Ventosa, 2006; Ghai et al., 2011; Ventosa et al., 2014). A pure culture of *Hqr. walsbyi* strain HBSQ001 has already been the subject of a transcriptome analysis (Bolhuis et al., 2017) and is compared to a metatranscriptome analysis of the CR30 crystallizer inhabiting *Hqr. walsbyi* community that was sampled at two different seasons (summer and winter).

## Materials and methods

### Sampling

Five liters of water sample were collected in January 2012 (Winter Sample) and July 2014 (Summer Sample) from CR30, a crystallizer pond located in the multi-pond solar saltern Bras del Port (Santa Pola, Alicante, Spain, 38°11′43.0”N 0°35′30.7”W). A hand refractometer was used to measure the salinity which in winter was found to be 31.5% (315 g kg^−1^) at a brine temperature of 12°C and in summer the salinity was 37.9% (379 g kg^−1^) at a brine temperature of 27°C. Samples were sequentially filtered through 20, 5 μm pore size polycarbonate filter, and 0.22 μm pore size Sterivex filter (Durapore; polycarbonate filters (Millipore, Billerica, MA, United States) and immediately frozen on dry ice in an RNA-stabilizing agent (RNAlater® Solution; Ambion, United States). Samples were stored at −80°C until processing.

### RNA isolation and cDNA synthesis

RNA isolation and cDNA synthesis was largely performed as previously described (Kimes et al., 2014), see details below. Before total RNA extraction with the RNeasy® Mini Kit (Qiagen,) in accordance with the instruction from the manufacturer, filters were treated with 500 μl of TE (Tris–HCl 10 mM, EDTA 1 mM, pH 8.0) containing lysozyme (2 mg/ml) and proteinase K (0.4 mg/ml) for 10 min at room temperature. Residual genomic DNA was removed from the extracted RNA by DNAse I treatment (Sigma-Aldrich) for 30 min at room temperature. Agarose gel electrophoresis and staining confirmed the absence of genomic DNA from the extracted RNA. Total RNA (10 μg) was used to make single-stranded cDNA using the High-Capacity cDNA Reverse Transcription kit (Applied Biosystems) following the manufacturer’s instructions. The second strand was synthesized by adding 30 units (U) of *Escherichia coli* Polymerase I (New England Biolabs), 5 U of *E. coli* DNA Ligase (New England Biolabs), 5 U of RNase H (Epicentre), 300 μM of dNTPs (Invitrogen) to the first strand reaction. After 2.5 h at 16°C, 5 U of T4-DNA polymerase (New England Biolabs) was added and incubated for 40 min at 16°C. Finally, the double-stranded cDNA was cleaned with a QIAquick PCR Purification kit (Qiagen) and quantified using the ND-1000 Spectrophotometer (NanoDrop, Wilmington, United States). The quality of all cDNA samples was determined on an Agilent 2,100 bioanalyzer. Paired-end sequencing of the cDNA was performed at EBI using half a lane of Illumina HiSeq2000 with 100 cycles per run (PE100).

### Metatranscriptome analysis

Reads from metatranscriptomes were pre-processed by Trimmomatic (Bolger et al., 2014) in order to remove low-quality bases (minimum Phred-quality score of 20 in a 4-base sliding windows) and reads shorter than 40 bases. The software package EMIRGE (Miller et al., 2011) was used to reconstruct full-length small subunit ribosomal genes and to provide length-normalized estimation the relative abundances. The assembled 16S rRNA sequences were clustered at 97% identity using the software cd-hit (Li and Godzik, 2006) and annotated against the SILVA version 128 reference database (Pruesse et al., 2007). Trimmed sequences were mapped against *Hqr. walsbyi* HBSQ001 (chromosome NC_008282.1, plasmid NC_008213.1) using edge-pro (Magoc et al., 2013). The number of reads aligned on each gene were counted and normalized to give TPM values (transcripts per million) (Wagner et al., 2012). Transcriptomic reads were analyzed and functional annotated using GhostKOALA:KEGG tools (Kanehisa et al., 2016). Transcriptomic reads from cultured *Hqr. walsbyi* HBSQ001 (Bolhuis et al., 2017) were analyzed following the same pipeline. Metatranscriptome reads from the winter and summer sample were deposited at the NCBI SRA sequence read archive (https://www.ncbi.nlm.nih.gov/sra) under bioproject number PRJNA633445.

## Results

### Metatranscriptome characteristics

More than 27 million and 35 million reads were obtained for the summer and winter samples, respectively (Table 1). Respectively 21% (summer) and 13% (winter) were annotated as 16S rRNA coding reads. Assembly and taxonomic annotation of 16S and 18S ribosomal RNA genes using EMIRGE revealed 91.4% of archaeal ribosomal transcripts sequences in the summer samples and 77.1% in winter samples (Supplementary Dataset-1, Sheet-1). Bacterial derived 16S rRNA reads accounted for 8.4% of the total number in summer transcripts but were nearly 3-fold more abundant during winter (22.7%). The eukaryotic fraction, mainly constituted by the halophilic unicellular algae *Dunaliella* sp., remained stable at 0.2%. Most of the 16S rRNA reads were assigned to the genus of *Haloquadratum*, making up 55% of the total expressed rRNA genes in summer and 47% in winter. Other abundant active genera during the summer period were *Haloarcula* (12%) and *Salinibacter* (7%). The winter population revealed a 5-fold lower number of *Haloarcula* assigned reads (2%), while *Salinibacter* accounted for a relative increase up to the 16% of the total 16S-rRNA reads. Other haloarchaeal genera that were significantly present at more than 1% of in the summer community were identified as novel haloarchaeal genera (unclassified reads) together with *Halolamina*, *Natrialba*, *Halobellus*, *Halonotius*, *Halorubrum* and *Halosimplex*. With the exception of the novel haloarchaeal genera, all of these had a lower abundance in the winter sample, especially *Halolamina* and *Natrialba*, which were 18-fold and 11-fold less abundant, respectively.

**Table 1:** General statistics of the winter and summer metatranscriptomes and reads recruited to *Hqr. walsbyi* HBSQ001.

	Summer	Winter
Total number of reads	27.765.941	35.609.827
% *Hqr. walsbyi* recruited reads	31%	25%
% *Hqr. walsbyi* reads assigned as mRNA	28% (8.7% of total)	40% (10% of total)
Number of genes covered^1^	2.614	2.616
Average read number per gene	884	1.355
Fold coverage per gene	74	109

^1^Only genes were included that recruited at least 10 reads per gene.

### Difference in gene expression patterns between natural and culture grown *Hqr. walsbyi* HBSQ001

**Figure 1:**
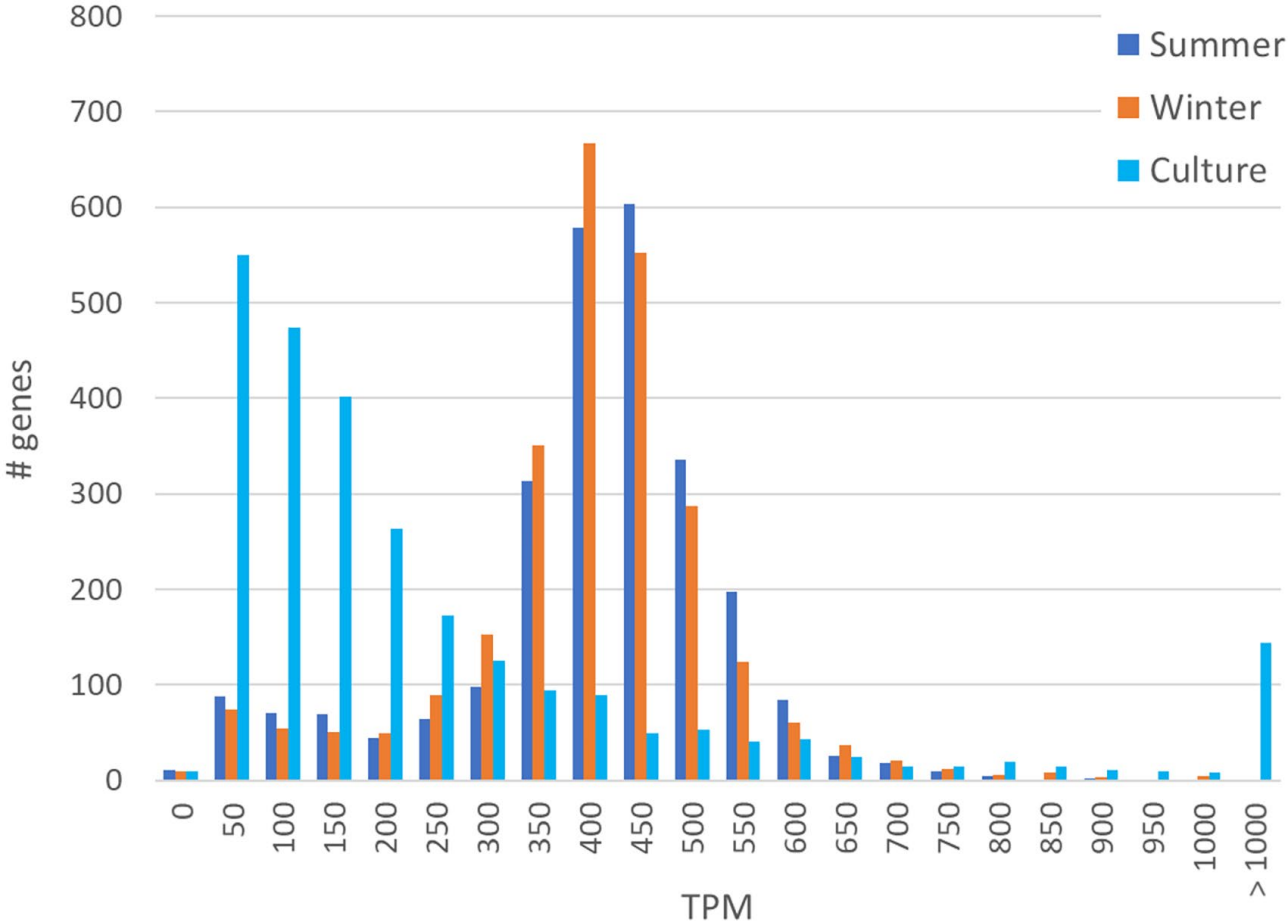
Histogram of TPM distribution and number of genes involved in the two metatranscriptomes (summer and winter) in comparison to the transcriptome of the cultivated isolate *Hqr. Walsbyi* HBSQ001.

Comparison of the distribution of the normalized number of transcripts per gene (TPM) revealed a clear distinction between the culture transcriptome and environmental metatranscriptome. The TPM distribution in the summer and winter derived metatranscriptomes follow a bell-shape curve with a median of 399 TPM (summer) and 387 TPM (winter), close to the genome average expression of 381 TPM (Figure 1). In the culture transcriptome however, the histogram reveals that most genes are low expression (around 50 to 150 TPM) with a median of 132 TPM whereas a small number of genes (144) have expression levels well above 1,000 TPM. Many genes that are low expressed in culture but significantly expressed in nature are uncharacterized, hypothetical proteins and IS elements. An interesting example is a small, ~10Kb, region of genes mostly encoding hypothetical proteins (HQ_RS07905 - HQ_RS07945) that are expressed in nature but not, or barely, in culture (Supplementary Dataset-1, Sheet-2). This region includes the potential L-lactate permease (*lctP*) that revealed an average expression in both metatranscriptomes (377 TPM) while not a single read was assigned to this gene in the cultured strain. Amongst the genes that are overexpressed in the culture but low expressed in nature, there is a 1,626 amino acid cell surface adhesin protein coding gene (HQ_RS01080; 7736 TPM in culture and <100 TPM in nature), two genes encoding uncharacterized proteins, HQ_RS11710 and HQ_RS12980 (respectively 827 and 818 TPM in culture versus <7 TPM in nature) and two genes, *neuA* and *neuB*, involved in sialic acid synthesis (both only expressed in culture but not in nature). Genes that are expressed significantly higher in nature than in culture include ABC-type transporters involved in transport of macrolides, nickel or nitrates and several major facilitator superfamily transport proteins for unknown substrates. The *coxMSL* gene cluster is between 13 and 27-fold higher expressed in nature than in culture.

### Seasonal difference in gene expression patterns

Normalized average transcription for 2,625 mRNA coding genes is 381 TPM. The genome of *Hqr. walsbyi* HBSQ001 recruited 31% of the total number of reads from the summer sample and 25% from the winter sample. Messenger RNA genes accounted for 28% (summer) and 40% (winter) of the *Hqr. walsbyi* recruited reads and recovered 2,614 and 2,616 genes of the previously 2,625 identified genes on the genome (Table 1). The average number of reads per gene was 884 (summer) and 1355 (winter) with an estimated 74- and 109-fold coverage per gene.

**Figure 2:**
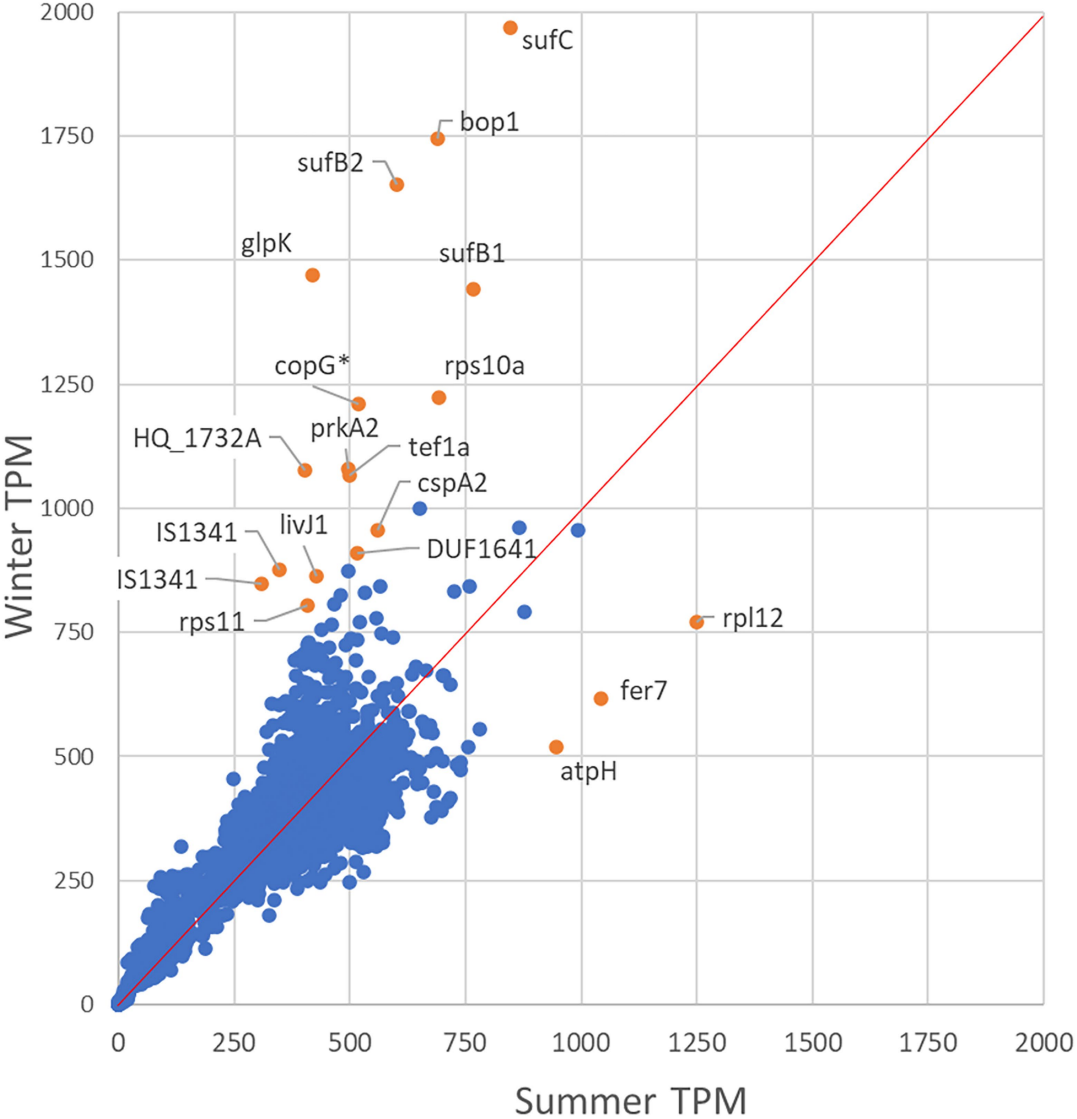
Differential expression analysis. The scatter plot shows the gene expression level in the summer (x-axis) versus the winter population (y-axis). Genes below the 1 tot 1 expression line are higher expressed in summer and above the 1 to 1 line are higher expressed in winter. Significantly differentially expressed genes are indicated in orange.

Comparison of TPM normalized gene expression levels in *Hqr. walsbyi* HBSQ001 between summer and winter derived metatranscriptomes (Figure 2) revealed that out of the 2,625 identified genes, 1,136 genes had a similar expression level (defined as a difference in TPM between summer and winter less than one tenth of the average expression: 38 TPM). In total, 795 genes were higher expressed in summer than in winter and 694 genes were higher expressed in winter relative to the summer (Supplementary Dataset-1, Sheet-3). Significant seasonal different expression was established for genes that had a combined expression (summer + winter) of at least 76 TPM (2 times one tenth of the average) and at least 1.5-fold difference in expression between summer and winter. This resulted in 195 differentially expressed genes, 55 of which were higher expressed in summer and 140 were higher expressed in winter (Supplementary Dataset-1, Sheet-3).

Out of the 55 genes that were higher expressed in summer than in winter, 37 are annotated as hypothetical- or uncharacterized protein coding genes. Summer high expressed genes with predicted functions encode an A-type ATPase subunit H (*atpH*), a Sec-independent translocase (*tatA*), ferredoxin proteins (*fer7* and *fer2*) and three thioredoxin proteins (*trxA6*, *trxA1* and *trxA5*). Out of the 140 genes that were higher expressed in winter than in summer, 14 genes were found to be at least 2.5-fold higher expressed than in summer. Highest differential expression (3.5-fold) was found for the glycerol-kinase encoding gene *glpK*. The gene HQ_RS03760, immediately upstream of *glpK* and well conserved amongst halophilic archaea, was also highly expressed in winter. Genes encoding the dihydroxyacetone PTS system are above average expressed and the dihydroxyacetone kinase subunit-L coding gene (*dhaL)* was significant higher (1.58-fold) expressed in winter than in summer. Other highly differentially expressed genes in winter were *sufB2*, *sufC* and *sufB1* (encoding ferredoxin assembly proteins), *bopI* (encoding the proton translocating bacteriorhodopsin) and *uraA2* (encoding a xanthine/uracil permease). Only 18.5% of the genes encoded for hypothetical or uncharacterized proteins. Furthermore, the winter sample revealed a higher expression of genes encoding the A-type ATPase subunit F (*atpF*), the gas-vesicle protein A (*gvpA*) and the NADH dehydrogenase-like complex subunits A and B (*nuoA* and *nuoB*).

### Overall gene expression levels

In addition to seasonal differences in expression, we also looked at the relative expression levels in both samples. Protein coding genes were considered highly expressed if their expression level is at least 1.5 times higher than the average expression (1.5 × 381 TPM = 571 TPM). In the summer sample, 99 genes were found to be significantly higher expressed and, in winter, 134 genes were expressed above the average. Among these, there are 30 genes that were found highly expressed in both samples. The top twenty-five protein-coding genes in summer and winter are listed in Tables 2, 3 respectively. In summer, the top twenty-five contains five yet uncharacterized proteins and includes gene HQ_RS01075, the overall highest expressed gene in both the summer and winter sample (Table 2). Also highly expressed in summer were the 50S and 30S ribosomal protein coding genes, three IS transposases, genes involved in ferredoxin assembly (*fer7, sufC* and *sufB1*), thiosulfate sulfurtransferase and thioredoxin biosynthesis. In winter, the top twenty-five, headed by HQ_RS01075, also contains an operon with genes involved in ferredoxin assembly (*sufC, sufB1* and *sufB2*), the same two 30S ribosomal protein coding genes as found in summer, IS transposases (two of them were also detected in the summer top 25) and uncharacterized protein coding genes (Table 3). The genes *bop1* and *glpK* were found at the third and fifth most abundant position, respectively. Nine out of the 25 highest expressed genes in summer were also found in the top 25 of the winter sample (Tables 2, 3, bold). Among them, there are several genes involved in stress response; i.e., the thermosome subunits Ths1 and Ths2, the cold shock proteins CspA1 and CspA2, the ATP-dependent protease Lon and the superoxide dismutase Sod.

**Table 2:** Top 25 of highest expressed protein coding genes in the summer population of *Hqr. walsbyi* HBSQ001.

Gene ID	TPM	*gene*	Annotation
**HQ_RS01075**	**2,380**	***-***	**Uncharacterized protein**
HQ_RS09700	1,251	*rpl12*	50S ribosomal protein L12
HQ_RS12535	1,043	*fer7*	Ferredoxin (2Fe-2S)
**HQ_RS13010**	**993**	***-***	**ISH8-type transposase ISHwa8**
HQ_RS11475	945	*atpH*	A-type ATP synthase subunit H
HQ_RS09370	878	*rpl29*	50S ribosomal protein L29
**HQ_RS05300**	**866**	***-***	**ISH9-type transposase ISHwa1**
**HQ_RS03630**	**847**	***sufC***	**Fe-S cluster assembly ATPase SufC**
HQ_RS08655	780	*-*	CopG domain protein
**HQ_RS03635**	**767**	***sufB1***	**SufB domain protein**
**HQ_RS09925**	**759**	***rps13***	**30S ribosomal protein S13**
HQ_RS13890	755	*tssA2*	Thiosulfate sulfurtransferase
HQ_RS11220	740	*-*	Uncharacterized protein
HQ_RS01670	738	*trxA1*	Thioredoxin
HQ_RS02195	732	*-*	Uncharacterized protein
**HQ_RS01400**	**725**	***cspA1***	**Cold shock protein**
HQ_RS09285	716	*-*	Uncharacterized protein
HQ_RS12580	716	*-*	Uncharacterized protein
HQ_RS06655	712	*-*	Cupin 2 barrel domain protein
HQ_RS09630	704	*rpl8e*	50S ribosomal protein L8e
HQ_RS04415	701	*cspA3*	Cold shock protein
HQ_RS07460	700	*-*	ISHwa2-type transposase ISHwa2
HQ_RS07640	698	*tatA*	Sec-independent protein translocase subunit TatA
**HQ_RS12175**	**692**	***rps10a***	**30S ribosomal protein S10a**
**HQ_RS00075**	**689**	***bop1***	**Bacteriorhodopsin I**

Genes in bold indicated genes that are highly expresses in both summer and winter.

**Table 3:** Top 25 of highest expressed protein coding genes in the winter population of *Hqr. walsbyi* HBSQ001.

Gene ID	TPM	*gene*	Annotation
**HQ_RS01075**	**2,380**	***-***	**Uncharacterized protein**
**HQ_RS03630**	**847**	***sufC***	**Fe-S cluster assembly ATPase SufC**
**HQ_RS00075**	**689**	***bop1***	**Bacteriorhodopsin I**
HQ_RS03640	601	*sufB2*	SufB domain protein
HQ_RS03765	419	*glpK*	Glycerol kinase
**HQ_RS03635**	**767**	***sufB1***	**SufB domain protein**
**HQ_RS12175**	**692**	***rps10a***	**30S ribosomal protein S10a**
HQ_RS02125	519	*-*	CopG domain protein
HQ_RS10230	496	*prkA2*	Probable PrkA-type serine/threonine protein kinase
HQ_RS03760	404	*-*	Uncharacterized protein
HQ_RS12180	500	*tef1a*	Translation elongation factor aEF-1 alpha subunit
HQ_RS01950	651	*-*	Uncharacterized protein
**HQ_RS05300**	**866**	***-***	**ISH9-type transposase ISHwa1**
**HQ_RS13010**	**993**	***-***	**ISH8-type transposase ISHwa8**
HQ_RS02000	560	*cspA2*	Cold shock protein
HQ_RS00700	515	*-*	DUF1641 family protein
HQ_RS05510	347	*-*	IS1341-type transposase
HQ_RS02130	498	*ftsZ2*	Cell division protein FtsZ, type II
HQ_RS03190	428	*livJ1*	ABC-type transport periplasmic substrate-binding protein
HQ_RS01835	310	*-*	IS1341-type transposase
HQ_RS07465	564	*sod*	Superoxide dismutase (Mn)
**HQ_RS09925**	**759**	***rps13***	**30S ribosomal protein S13**
**HQ_RS01400**	**725**	***cspA1***	**Cold shock protein**
HQ_RS05000	533	*aldH2*	Aldehyde dehydrogenase
HQ_RS00695	480	*-*	Probable anaerobic dehydrogenase alpha subunit

Genes in bold indicated genes that are highly expresses in both summer and winter.

**Figure 3:**
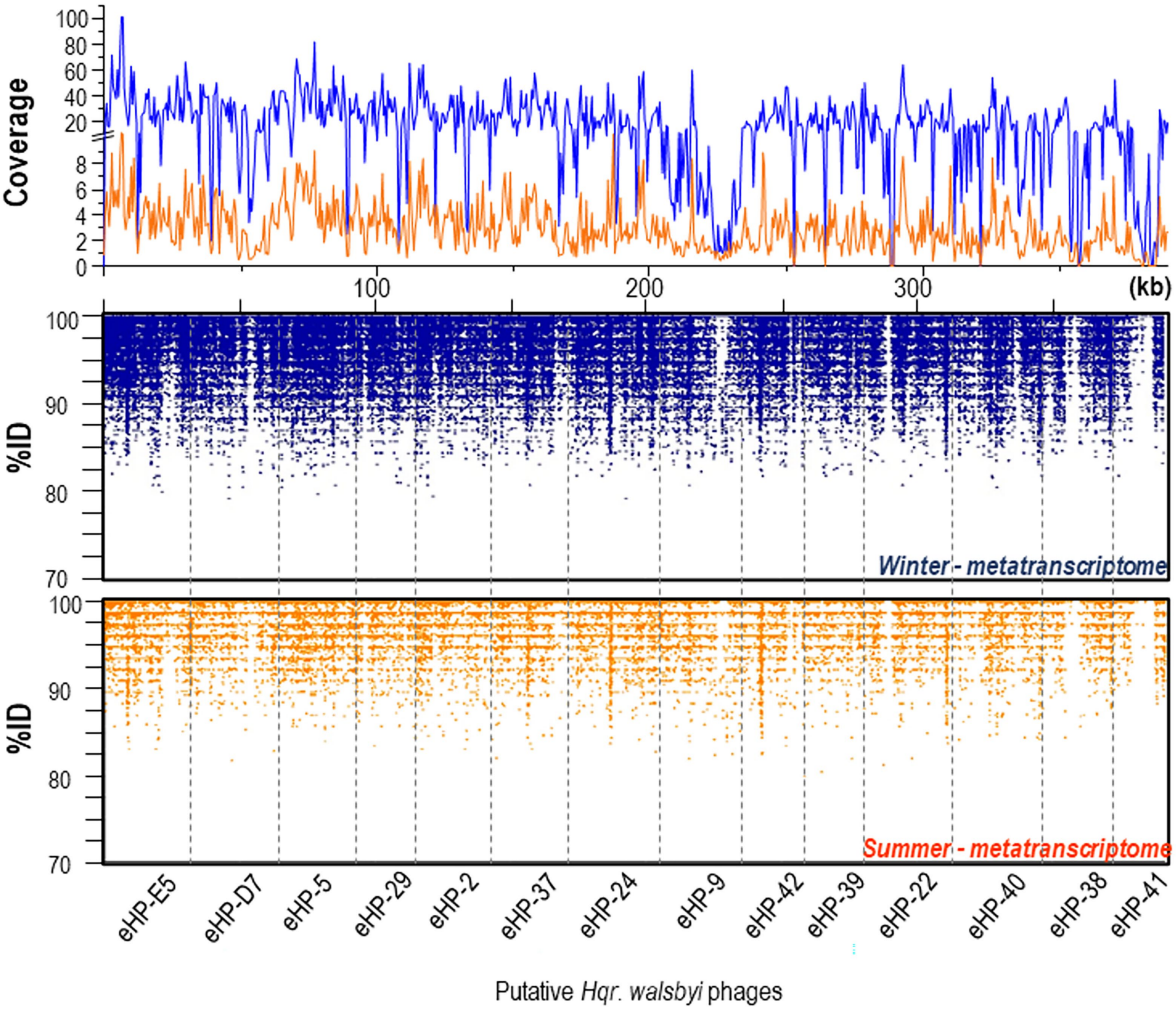
Recruitment plots of putative *Hqr. walsbyi* virus genomes (eHP-) against metatranscriptomic reads from summer (bottom panel) and winter (middle panel) samples. (Top panel) Coverage plot of the metatranscriptomic reads mapped onto the viral genes in summer (orange) and winter (blue).

We also analyzed expression levels of viral genes from the 14 *in-silico* assembled viral genomes assigned to *Hqr. walsbyi* based on the presence of CRISPR spacers (Garcia-Heredia et al., 2012). Curiously, the viral genes were almost one order of magnitude higher expressed in winter than in summer (Figure 3). Exceptions are eHP-9, eHP-41 and eHP-D7 that were barely or not over the full length recruited in the winter and summer samples.

Regarding the low expressed genes, the summer and winter samples contained, respectively, 11 and 9 genes that were not expressed at all, 8 of them were absent from both samples. These unexpressed genes mainly encode uncharacterized and hypothetical proteins, but also encode for acylneuraminate cytidylyltransferase (*neuA*) and sialic acid synthase (*neuB*). Notably, amongst the low expressed genes are several sulfatases, glycosyl transferases and extracellular glycoprotein coding genes including the two short-halomucin coding genes (*hmu2* and *hmu3*). The long-halomucin coding gene *hmu* is expressed at 0.6 (summer) and 0.7 (winter)-fold average.

### Genome expressed hot spots

Analysis of the genome localization of highly differentially expressed genes revealed three specific regions with a higher than average difference (1.22-fold) in gene expression between summer and winter (Supplementary Dataset-1, Sheet-4). The first region, with an average differential expression of 1.41, ranges from nucleotide position 849,423–900,876, and contains the above-described genes *sufB1*, *sufB2*, *sufC*, *glpK* and HQ_RS03760. The second region has an average differential expression of 1.41 and ranges from nucleotide position 1,270,976–1,345,673. It partly overlaps with one of the six previously identified genomic islands (Cuadros-Orellana et al., 2007), HQ_GI2, and encodes mainly hypothetical and uncharacterized proteins. Three genes encode known proteins and are involved in nucleotide metabolism; i.e. a xanthine/uracil permease family transport gene protein (*uraA2*; 2.48-fold), a purine phosphoribosyltransferase (*apt2*; 1.68-fold), and a signal transduction histidine kinase (HQ_RS14555; 1.90-fold). The third region is found at the end of the genome (2,867,030 to 2,981,278) and partly overlapping with genomic island HQ_GI4. It contains 51 genes, and, with 1.86-fold difference, it was the highest differential expressed region by far. Only one these 51 genes was found to be higher expressed in summer and encodes a hypothetical protein (HQ_RS12985; 1.85-fold difference). High expressed genes in this region consist of several genes encoding toxin/antitoxin systems such as NUDIX family hydrolase, HicB family protein, AbrB/VapB family protein, and copies of antitoxin VapB and VapC.

In general, gene expression levels in each island is lower than the genome average. The only exception was represented by HQ_GI1, mainly because the overall highest expressed gene HQ_RS01075 is located on this island (Supplementary Dataset-1, Sheet-5). The average differential gene expression per genomic island between winter and summer was close to the genome average in HQ_G1 (1.25), HQ_G1b (1.27) and HQ_G2 (1.28), and relatively higher in HQ_GI2b (1.47), HQ_GI3 (1.50) and HQ_GI4 (1.58).

### *Hqr. walsbyi* HBSQ001 ecoparalogs

*Hqr. walsbyi* HBSQ001 contains several potential ecoparalogs, i.e., genes encoding similar proteins (paralogs) that may be differentially expressed under different environmental conditions (Sanchez-Perez et al., 2008). Paralogous genes that differed significantly (>1.5-fold) in expression amongst each other within the same sample qualified as ecoparalogs. We identified 150 genes, grouped in 63 clusters that can be considered as candidate “ecoparalogs” (Supplementary Dataset-1, Sheet-6). In total, 31 clusters with differentially expressed genes were found in summer and 27 in winter. Highest differences in expression between ecoparalogs were found for the uncharacterized proteins HQ_RS05860 and HQ_RS13150 (8.8-fold) in summer and for ISH8 transposases (6.1-fold) in the winter dataset. To see whether seasonality may be a driver of ecoparalog differential expression, we compared their expression between the two samples. Three seasonal-ecoparalogs could be found, two ferredoxin coding genes, *fer2* and *fer7,* and a purine phosphoribosyltransferase coding gene, *apt2*, which had 1.5-fold, 1.7-fold and 1.7-fold higher expression in summer than in winter, respectively. We also calculated the difference between the highest expressed gene in a cluster from one season divided by the lowest expressed gene in that cluster from the other season. In 19 clusters the significant higher expressed gene was found in summer and in 33 clusters in winter. The highest differential expression in summer was found for the above mentioned ISH8 transposase coding genes (6.3-fold difference) and in winter for the uncharacterized proteins HQ_RS05860 and HQ_RS13150 (10-fold) coding genes.

## Discussion

The presented metatranscriptome analysis of the crystallizer ponds in summer and winter confirmed the dominance of halophilic Archaea at the highest salinities, and especially of *Hqr. walsbyi*. In summer, Bacteria and Eukarya do not exceed 10% of the total read abundance while in winter, their relative contribution increases to over 20%. At this moment, it is unclear whether this is a consequence of on average lower temperatures, less sun hours and lower sun intensity or whether the nutritional status of the brines dramatically change in winter.

The highest expressed *Hqr. walsbyi* HBSQ001 gene in both summer and winter sample is HQ_RS01075, which encodes a 72 amino acid small protein with a putative DNA binding motive (ribbon-helix–helix) found in the CopG-like protein family IPR002145, suggesting a role in regulating DNA transcription. Interestingly, this gene is absent from the Australian type strain, *Hqr. walsbyi* C23^T^ (Burns et al., 2007). Moreover, 100% identical homologs of this protein have so far only be found in the salterns from which strain HBSQ001 was isolated and may hint to a local evolved or acquired gene. Among the highly expressed genes in winter, several encode stress response proteins like cold-shock proteins, starvation-induced proteins, and superoxide dismutases. However, only a few of these differed significantly in expression compared to summer, suggesting that winter conditions alone, did not induce this stress response. Apparently, these genes are in a continuous high expression state and may reflect the year-round challenging conditions in the hypersaline ecosystem, such as high salinity, light intensities and UV irradiation, high temperatures and high oxygen radical concentrations (Salin and Brown-Peterson, 1993). Although a connection between these proteins and viral infection is not clear, viruses could be an important factor to explain specific rises of these stress-related proteins in *Hqr. walsbyi*. Two possible interpretations are, (i) that the total *Hqr. walsbyi* population is more susceptible to viruses in a more stressed winter environment, or, alternatively, (ii) that a specific dominant *Hqr. walsbyi* winter-ecotype is more susceptible to these viruses.

The winter high *bopI* expression may reflect a lower energy and nutritional status due to reduced photosynthetic primary production driven by algae like *Dunaliella*. Increased expression of *bopI* may also compensate for less and lower intensity sun hours. High expression of *sufB* and *sufC* in winter may be essential to meet an increased demand for electron translocating ferredoxins or for Fe-S proteins involved in distribution of electrons to various metabolic pathways. Higher expression of the A-type ATPase subunit F coding gene *atpF* and the NADH dehydrogenase-like complex subunits A and B, encoded by *nuoA* and *nuoB*, also agree with an increased need for energy requiring processes. Potential carbon limitation in winter may be deduced from high *glpK* gene expression. A higher glycerol demand in winter is further substantiated by the high expression of the hypothetical protein encoding-gene HQ_RS03760, directly upstream of *glpK*. This gene has a conserved PRK10712 domain, found also in the PTS system fructose-specific transporter subunits IIBC. Potentially, this protein might be involved in the uptake of glycerol rather than fructose and may form a phosphotransferase system with GlpK. The well-established PTS system involved in dihydroxyacetone uptake (Bolhuis et al., 2006; Elevi Bardavid and Oren, 2008; Ouellette et al., 2013) is above average expressed, while the uptake kinase of this system, encoded by *dhaL* is significantly higher expressed in winter.

### Evidence for multiple occurring ecotypes of *Hqr. walsbyi*

Potential hotspots for differentially expressed genes relative to the cultured isolate are two of the so-called genomic islands. In contrast to the well conserved core genome, these genomic islands represent regions in the genome that are not conserved amongst all members of the population. Therefore, they might be indicative for the presence of locally adapted ecotypes (Rodriguez-Valera et al., 2016). These islands can be identified by metagenomic recruitment analyzes against the genome of a cultivated strain. The core genome will recruit high numbers of near identical reads whereas the genomic islands will only have a few reads and often with low identity. For *Hqr. Walsbyi* HBSQ001, six of these putative genomic islands were initially identified (Cuadros-Orellana et al., 2007). Later, genome comparison between the two sequenced isolates of *Hqr. walsbyi*, strain HBSQ001 and strain C23^T^, suggested the presence of at least 12 of these divergent regions (Cuadros-Orellana et al., 2007; Dyall-Smith et al., 2011). It is believed that the content of these genomic islands can vary per ecotype and may provide specific, selective advantages for their particular ecological niche (López-Pérez et al., 2014; Rodriguez-Valera et al., 2016). The genomic islands in *Hqr. walsbyi* predominantly contained genes involved in biosynthesis of surface layer proteins, genes encoding cell surface glycoproteins and genes involved in the cell envelope formation (Martin-Cuadrado et al., 2015). The overall low gene expression levels within these islands agree with their genes being only present in a smaller subset of the *Hqr. walsbyi* population and hence the existence of different ecotypes that may be more abundant than the isolated strain. Presence of *Hqr. walsbyi* C23^T^ in the Spanish salterns was also investigated. A distinction between C23^T^ and HBSQ001 is difficult to make based on the core genome that share over 98% nucleotide identity (Dyall-Smith et al., 2011). However, absence of transcripts mapping to the genomic islands specific to C23^T^ is in agreement with the absence of this Australian isolate from the Spanish salterns.

In the cultured strain HBSQ001, these islands were also low expressed, but again, with exception of HQ_GI1, where the averaged expression level was 18-fold higher than the genome average. This is mainly caused by the presence of some of the overall highest expressed genes; the potential transcriptional regulator HQ_RS01075 and three cell surface glycoprotein coding genes, HQ_RS01080, HQ_RS01085 and HQ_RS01090. This suggests a clear difference between growth of *Hqr. walsbyi* in nature or culture. In culture, the cell surface glycoprotein coding genes are over expressed while in nature, the focus appears to be more on energy and carbon acquisition. This may be a consequence of the different geochemical composition of the culture medium compared to the natural brine which may have higher diversity in carbon sources at lower concentrations. Noticeable is also that majority of genes of the Islands HQ_GI2 and HQ_GI4 are higher expressed in nature than in culture and in HQ_GI2b, where 10 out of 12 genes are higher expressed in culture. Moreover, in HQ_GI1, HQ_GI1b, HQ_GI2b and HQ_GI4, more than 70% of the genes that are higher expressed in culture are also highest expressed in winter. Potentially, the isolated strain HBSQ001 or closely related ecotypes are more abundant in the winter season.

### Difference in expression patterns between natural and culture grown *Hqr. walsbyi* HBSQ001

One of the discussions in experimental microbial ecology concerns the validity of extrapolating gene expression patterns obtained in lab grown cultures to the natural environment. We therefore compared the metatranscriptome dataset with the transcriptome from *Hqr. walsbyi* HBSQ001 grown in a defined medium (Bolhuis et al., 2017). The most remarkable and noteworthy difference in gene expression pattern is the difference in TPM distribution per gene. The transcript distribution in the natural environment followed a bell-shape curve with a median close to the average TPM suggesting that most genes in nature are expressed close to the average. In culture however the median was much lower than the average and revealed that most genes were low expressed with the exception of a small number of very high expressed genes. This most likely reflects the adaptation of the cultured strain to the relative homogeneous, simple growth conditions and media composition. In nature, *Hqr. walsbyi* is represented by multiple ecotypes, adapted to heterogeneous environmental conditions in terms of temperature, light, UV irradiation and salinity. In addition, they are in continuous competition for the available nutrients and under potential threat of viral attack either with other halophilic species or even amongst their different ecotypes. Under laboratory conditions, several naturally occurring stressors may be absent. A good example is the expression of a gene encoding an L-lactate permease involved in the uptake of lactase that is well expressed in nature but not in culture. Lactate was not present in the cultivation medium but is a natural occurring carbon source in salterns and requires active transported over the membrane for uptake (Oren and Gurevich, 1994). One intriguing observation is the higher expression of the *coxMSL* gene cluster in nature. These genes are annotated as subunits of the aerobic-type carbon monoxide dehydrogenase; however, there is currently no evidence that carbon monoxide can be used as carbon source by *Hqr. walsbyi*. Potentially the true substrate of this molybdopterin binding dehydrogenase may be something else (King and Weber, 2007).

Genes that were overexpressed in culture but low expressed in nature include surface proteins and surface modification proteins that may be expressed in response to the limited movement in the standing batch cultures and may favor formation of biofilms and the much larger cell structures that were observed in the cultures compared to naturally occurring cells (Bolhuis et al., 2004).

## Conclusion

Our results clearly show that gene expression patterns in laboratory grown cultures do not necessarily reflect their metabolism in nature and therefore, extrapolating data from laboratory experiments to natural behavior should be done with care. Our results furthermore shows that there are multiple ecotypes of *Hqr. walsbyi* that differ from the HBSQ001 isolate. Low expression of this isolated strain specific genomic islands furthermore suggests that HBSQ001 isolate is not abundantly present in the pond and season from which it was originally isolated. In winter, the system appears more energy and carbon deprived and HBSQ001 is more abundant and hence may also be more virus tolerant, a feature which may have also led to its successful isolation. The differences in gene expression between natural occurring species and cultivated equivalents most likely reflect a higher number of available niches, presence of different nutrients and potential interactions with different species in the natural brine compare to the homogenous glycerol and pyruvate fed monocultures.

## Data availability statement

The datasets presented in this study can be found in online repositories. The names of the repository/repositories and accession number(s) can be found at: https://www.ncbi.nlm.nih.gov/, PRJNA633445.

## Author contributions

ML-P, AM-C, RR, and FR-V: conceived and designed the experiments. ML-P and RR: performed the experiments. RR, ML-P, AM-C, and HB: analyzed the data. RR, ML-P, AM-C, FR-V, and HB: wrote the paper. All authors contributed to the article and approved the submitted version.

## Funding

RR received funding from the European Union’s Horizon 2020 research and innovation programme under grant agreement no. 818431 (SIMBA). This output reflects only the author’s view, and the European Union cannot be held responsible for any use that may be made of the information contained therein. FRV was supported by grants “VIREVO” CGL2016-76273-P [MCI/AEI/FEDER, EU] (cofounded with FEDER funds) from the Spanish Ministerio de Ciencia e Innovación and “HIDRAS3” PROMETEU/2019/009 from Generalitat Valenciana.

## Conflict of interest

The authors declare that the research was conducted in the absence of any commercial or financial relationships that could be construed as a potential conflict of interest.

## Publisher’s note

All claims expressed in this article are solely those of the authors and do not necessarily represent those of their affiliated organizations, or those of the publisher, the editors and the reviewers. Any product that may be evaluated in this article, or claim that may be made by its manufacturer, is not guaranteed or endorsed by the publisher.

## Supplementary material

The Supplementary material for this article can be found online at: https://www.frontiersin.org/articles/10.3389/fmicb.2022.1044446/full#supplementary-material
